# Comparative Analysis of Cuticular Wax in Various Grape Cultivars During Berry Development and After Storage

**DOI:** 10.3389/fnut.2021.817796

**Published:** 2021-12-28

**Authors:** Mengwei Zhang, Peian Zhang, Suwen Lu, Qixia Ou-yang, Yaxian Zhu-ge, Ruiping Tian, Haifeng Jia, Jinggui Fang

**Affiliations:** ^1^College of Horticulture, Nanjing Agricultural University, Nanjing, China; ^2^College of Landscape Architecture, Nanjing Forestry University, Nanjing, China; ^3^State Key Laboratory of Crop Genetics and Germplasm Enhancement, Nanjing Agricultural University, Nanjing, China

**Keywords:** grape (Vitis vinifera), cuticular wax, crystal morphology, chemical composition, gene expression, berry development, post-harvest storage

## Abstract

Cuticular wax covering the surface of fleshy fruit is closely related to fruit glossiness, development, and post-harvest storage quality. However, the information about formation characteristics and molecular mechanisms of cuticular wax in grape berry is limited. In this study, crystal morphology, chemical composition, and gene expression of cuticular wax in grape berry were comprehensively investigated. Morphological analysis revealed high density of irregular lamellar crystal structures, which were correlated with the glaucous appearances of grape berry. Compositional analysis showed that the dominant wax compounds were triterpenoids, while the most diverse were alkanes. The amounts of triterpenoids declined sharply after véraison, while those of other compounds maintained nearly constant throughout the berry development. The amounts of each wax compounds varied among different cultivars and showed no correlation with berry skin colors. Moreover, the expression profiles of related genes were in accordance with the accumulation of wax compounds. Further investigation revealed the contribution of cuticular wax to the water preservation capacity during storage. These findings not only facilitate a better understanding of the characteristics of cuticular wax, but also shed light on the molecular basis of wax biosynthesis in grape.

## Highlights

- The high density of irregular lamellar crystal structures was correlated with the glaucous appearances of grape berry.
- The dominant wax compounds were triterpenoids, while the most diverse were alkanes in grape berry.
- The amounts of total wax and the expression levels of most related genes were significantly downregulated after véraison.
- The amounts of each wax compounds varied among different cultivars and showed no correlation with berry skin colors.
- Cuticular wax contributed to the water preservation capacity of grape berries.

## Introduction

Grape (Vitis vinifera) is an important fruit crop that is commonly grown worldwide (1). Cuticular wax is responsible for the whitish (glaucous) or glossy covering on the fruit, affecting the appearance quality and the market value of grape (2). Moreover, the hydrophobic properties of the cuticular wax have important roles in reducing non-stomatal water loss, protecting against ultraviolet radiation, and preventing pathogenic attacks during fruit development and storage (3). The grape berry skin is impregnated with aliphatic and cyclic wax compounds (4). The aliphatic fraction comprises a complex mixture of very long chain fatty acids (VLCFAs) and their derivatives, such as aldehydes, alkanes, ketones, alcohols, and alkyl esters. The cyclic fraction usually contains triterpenoids particularly of oleanolic acid and β-sitosterol, which exhibit numerous health beneficial properties including antioxidant, anticancer, anti-inflammatory, and anti-cardiovascular diseases (3, 5). Research interest has been focused on the cuticular wax in grape due to their important roles in fruit quality and post-harvest storage properties.

The biosynthesis pathway of cuticular wax has been elucidated in plants (6, 7). In general, the biosynthesis of aliphatic compounds of cuticular wax begins with C_16_ and C_18_ fatty acid synthesis in the plastids of epidermal cells. These molecules are then transported to the cytoplasm and elongated to VLCFAs (C_20_-C_34_) in the endoplasmic reticulum. VLCFAs are further modified to form various wax products, including alcohols, wax esters, aldehydes, alkanes, and ketones. A number of key genes, such as β*-ketoacyl-ACP synthase* (*KAS*), β*-ketoacyl-CoA synthase* (*KCS*), β*-ketoacyl-CoA reductase* (*KCR*), *enoyl-CoA reductase* (*ECR*, or *ECERIFERUM, CER*), and *bifunctional wax synthase/acyl-CoA:acyltransferase* (*WSD*) are involved in this process (7). Several transcription factors (TFs) are reported to regulate wax biosynthesis. These include APETALA2/ethylene responsive factor binding proteins WAX INDUCER1 (WIN1) and SHINE1 (SHN1) (8), ABA-mediated MYB protein MYB96 (9), and AP2/ERF protein DEWAX (10). Studies of cuticular wax biosynthesis have focused on some fruit crops, such as tomato (11), pear (12), bilberry (13), and citrus (14, 15), but research on grape berry is very limited.

Preliminary investigation of the composition of cuticular wax in grape berry were carried out as early as 1,892 and it has been found that oleanolic acid was the main component of grape berry cuticular waxes (3). Studies have revealed different crystal morphologies of cuticular wax including massive crusts, granules, plates, platelets, filaments, rods, and tubules with a hollow center (16). The composition and crystal morphologies of cuticular wax were influenced by a variety of factors including development stages, species, tissues, and environmental conditions (2, 4, 17). Previous studies have determined the wax composition in the berries of grape cultivars and identified several related genes, including CER and WIN1, in grape genome (3, 5, 18). However, our knowledge on the accumulation characteristics and molecular mechanisms of cuticular wax in grape is still fairly restricted.

Therefore, the objective of this study was to compare the morphological structure, the chemical composition, and the biosynthesis of cuticular wax in grape berry during development and among different cultivars. We also investigated the changes of cuticular wax amount and related gene expression during post-harvest storage. The production and composition evaluation of cuticular wax will provide important data on berry metabolism and its relation to quality attributes, with the ultimate goal being supporting grape fruit post-harvest quality.

## Materials and Methods

### Materials, Treatments, and Sampling

Grape berries were harvested from an orchard located in Jiangsu, China. Fruit samples from |Red Globe” (RG), “Kyoho” (KH), and “Shine Muscat” (SM) were collected at three developmental stages (S1, mature-green; S2, véraison; S3, maturation), respectively. Fruit samples from other nine cultivars (“Fujiminori,” FM; “Summer Black,” SB; “Wink,” WK; “Manicure Finger,” MF; “Queen of Vineyard,” QV; “Centennial Seedless,” CS; “Rosario Bianco,” RB; “Italia,” IT; “|Victoria,” VT) were collected at the commercial mature stage. After harvest, berries with normal shape, uniform size and color, no physical damages, and no diseases were randomly selected for further experiments. Berry development was tracked by measuring berry total soluble solids (TSS) of the collected samples.

“Kyoho” berries at the commercial mature stage were air dried and stored at two temperatures (4 and 25°C), and 80–90% relative humidity for 21 d. Berries were sampled at 0, 7, 14, and 21 d, respectively, and allowed to equilibrate at 20°C overnight at each sampling date. Thirty berries were divided randomly into three groups, and from which 10 berries were used to determine the rate of water loss. Then, 30 berries collected at 21 days after storage (DAS) were used for the microstructure analysis and cuticular wax extraction, and the remaining were used for the subsequent determination of gene expression.

In order to create little to no disturbance of the cuticular wax layer and avoid wiping of waxes from the berry surface during the sampling, berries were held with tweezers through the pedicel and carefully trimmed off the cluster with a pair of scissors. Partial berries were directly used for microscope observation and wax determination, and the remaining samples were immediately frozen in liquid nitrogen and stored at −80°C for gene expression analysis.

### Cryo-Scanning Electron Microscopy (Cryo-SEM)

Berry skin pieces (3 mm^3^) from the equatorial sections were excised using a blade, immediately frozen in liquid nitrogen (−210°C), and sent to the electron microscopy platform of Nanjing Agricultural University for subsequent manipulations and procedures. Observation of the ultrastructure of the epidermal wax crystals was carried out with a scanning electron microscope (HITA-CHI H-7650) equipped with a cryo preparation unit (Alto 2500, Gatan, UK).

### Extraction of Cuticular Wax

The cuticular wax was extracted according to the method as described by Wang et al. (15) with minor modifications. Three biological replicates were considered for each sample and each biological replicate consisted of a group of three vines, for a total of thirty berries. Grape berry was assumed to be perfect ellipsoid to calculate the fruit surface area. Fruit diameter values including a, b, and c were determined from the average of the vertical and horizontal diameters with a vernier caliper (mm). The surface area of the berry was estimated based on the calculation formula as follows: S (cm^2^) =4 / 3 × (a × b + a × c + b × c) × π /1000.

The entire berries were immersed twice in 10 mL of chloroform for 2 min at ambient temperature in a fume hood. The two chloroform extracts were combined, followed by adding 40 μL (5 μg/μL) of *n*-tetracosane as an internal standard. The combined extracts were added with 10 g Na_2_SO_4_ and mixed. The mixture was filtered through a three-layer filter paper. Five mL of the filtrate was taken and dried under a continuous flow of nitrogen; then dissolved in 300 μL of pyridine. After water bath for 30 min at 50°C, 300 μL of *bis*-*N, N*-(trimethylsilyl) trifluoroacetamide (BSTFA) was added. After derivatization for 40 min at 60°C, residual BSTFA and pyridine were blown dry under nitrogen and the samples were re-dissolved in 2 mL of chloroform. The solution was then filtered using a microporous film (0.22 μm) for further analysis by GC-MS. All solvents and reagents were of analytical grade in this research.

### Quantitative and Qualitative Analysis of Fruit Cuticular Waxes by Gas Chromatography—Mass Spectrometry (GC-MS)

GC-MS analysis was performed with a gas chromatograph (TRACE 1310, Thermo Scientific), coupled to a triple quadrupole mass spectrometer (TSQ 9000, Thermo Scientific). 1 μl of each sample was injected in split mode (ratio: 10) with about 9% of injected samples being transported by a carrier gas into a non-polar column (TG-5MS, 30 m, 0.25 mm ID, 0.25 μm film thickness, Thermo Scientific). The temperature of injector was 280°C. Helium at constant flow (1 mL/min) was used as the carrier gas. The GC used the following program: 50°C for 2 min; ramp 10°C min^−1^ to 200 °C; hold 5 min; ramp 5°C min^−1^ to 290 °C; hold 5 min; ramp 3°C min^−1^ to 320°C; hold 14 min. MS operating parameters were as follows: electron impact ionization, electron energy 70 eV, scan range 45–650 amu. Compounds were tentatively identified by mass spectrometry analysis: i.e., matching mass spectrum of samples with database in NIST 2017 mass spectral library. Single compounds were quantified against the internal standard and the amount was normalized to the surface area (μg/cm^2^). The amount of each compound in each sample was listed in Supplementary Table 1. The total amount of wax was calculated by summing up all fractions. The analysis was performed in triplicate.

### RNA Extraction, CDNA Synthesis, and Quantitative Real-Time PCR (qRT-PCR)

Total RNA was extracted using the traditional CTAB method. First-strand cDNA was synthesized from 1 μg total RNA using the Hieff First Strand cDNA Synthesis Super Mix (11103ES70, YEASEN). Gene-specific primers were designed by Primer Premier 5 (Supplementary Table 2) and subjected to qRT-PCR analysis as described previously. Each reaction was performed in triplicate and a negative water control was included in each run. The gene expression levels were normalized to the threshold cycle (Ct) value of the *ACTIN* gene and calculated according to the 2^−ΔΔCt^ method. The experiments were performed at least two times with similar results and representative data from one repetition were shown.

### Statistical Analysis

Statistical analysis was performed by one-way ANOVA of the SPSS program. Significant differences were indicated with different letters (*P* < 0.05).

## Results

### Crystal Morphology of Cuticular Wax in Grape Berry

**Figure 1:**
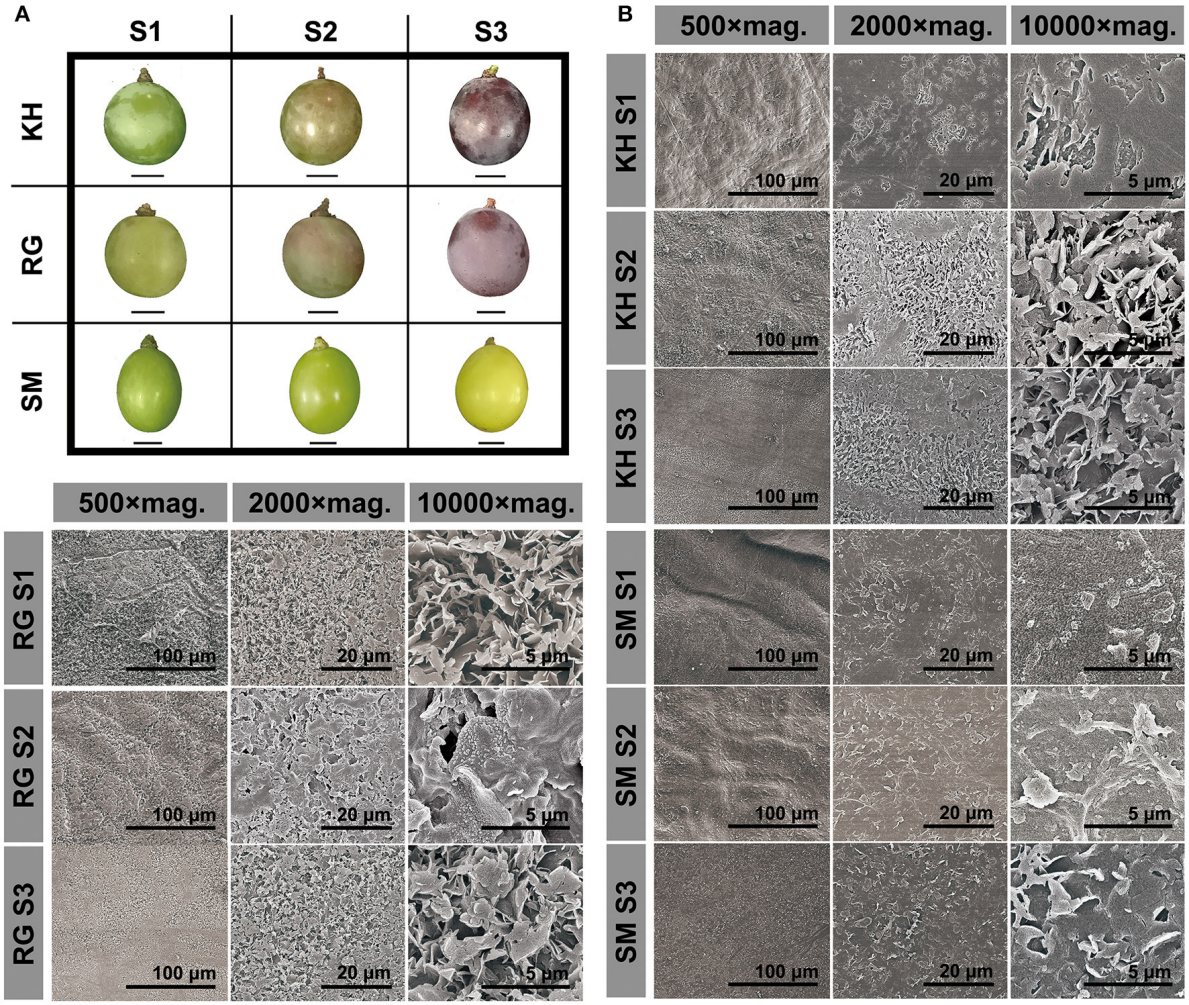
Berry morphology **(A)** and microstructure of cuticular wax **(B)** in three grape cultivars during berry development. Three grape cultivars: KH, “Kyoho”; RG, “Red Globe”; SM, “Shine Muscat”. Three developmental stages: S1, mature-green; S2, véraison; S3, maturation. mag. Magnification. Scale bars represent the magnification.

By visual inspection of the berry surface of three representative grape cultivars, the entire surface of “Kyoho” and “Red Globe” berries appeared to be coated with a gentle glaucous waxy layer and the white frost gradually thicken as the berry developed; while the surface of “Shine Muscat” berries appeared glossy phenotype throughout the development stages (Figure 1A). We further used Cryo-SEM to visualize the microstructure of cuticular wax during fruit development (Figure 1B). This tool enables to detect finest differences in the structure of the wax crystals which would completely disappear during the fixation process for SEM analysis. Cryo-SEM images showed that “Kyoho” and “Red Globe” had a dense lamellar crystal structure; and the wax crystals increased and clustered into large cracks on the sample surface with the berry development. Under a 500 × magnification, it was observed that the platelets almost covered the epidermal of berries. High magnification microscopic observation showed that the orientation of the platelets of different heights appeared random, resulting in a very irregular pattern. By contrast, the “Shine Muscat” berry surface was coated by a smooth wax film with markedly low density of crystalloid structures; the magnification of the crystal region revealed an amorphous layer of wax throughout the berry development.

### Chemical Composition and Amount of Cuticular Wax During Berry Development

**Figure 2:**
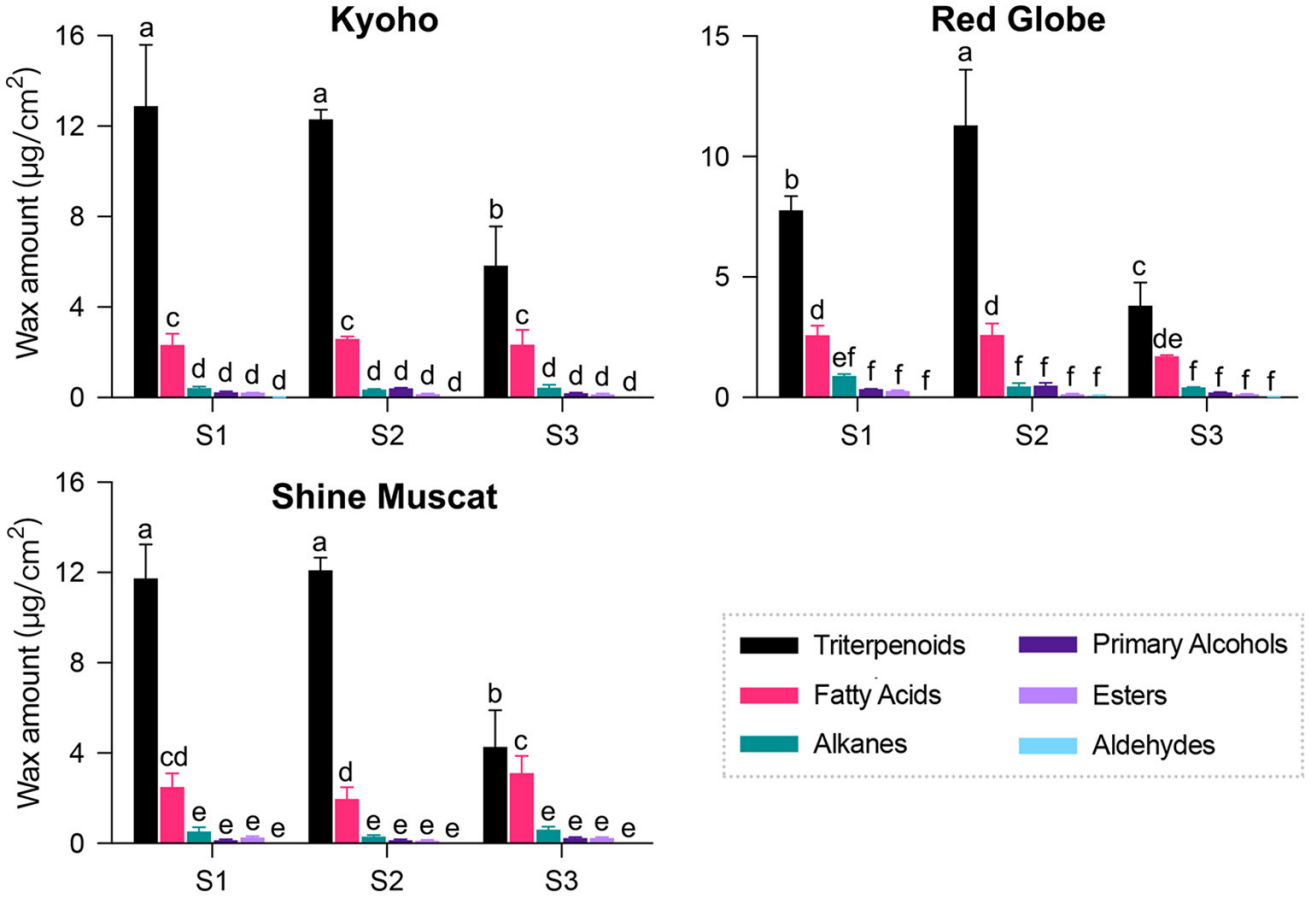
Composition and amount of cuticular wax in grape berries during development. Three developmental stages: S1, mature-green; S2, véraison; S3, maturation. Values are mean ± SD of three biological replicates. Different letters indicate significant differences at *P* < 0.05 (Student's *t*-test).

GC-MS analysis revealed that the components of cuticular wax in “Kyoho,” “Red Globe,” and “Shine Muscat” berries mainly included triterpenoids, fatty acids, alkanes, primary alcohols, esters, and aldehydes. The amount of each compound in each sample was listed in Supplementary Table 1. Triterpenoids followed by fatty acids were found to be the dominant compounds, while only traces of alkanes, primary alcohols, esters, and aldehydes were detected throughout the developmental stages. The amounts of triterpenoids were high at the early development stage, and declined sharply after véraison, while those of other compounds maintained nearly constant throughout the berry development. Changes in the amounts of each compound were similar among the three cultivars during development (Figure 2). These results indicate that cuticular waxes were biosynthesized at the early stage.

### Variation of Cuticular Wax Among Grape Cultivars With Different Skin Colors

**Figure 3:**
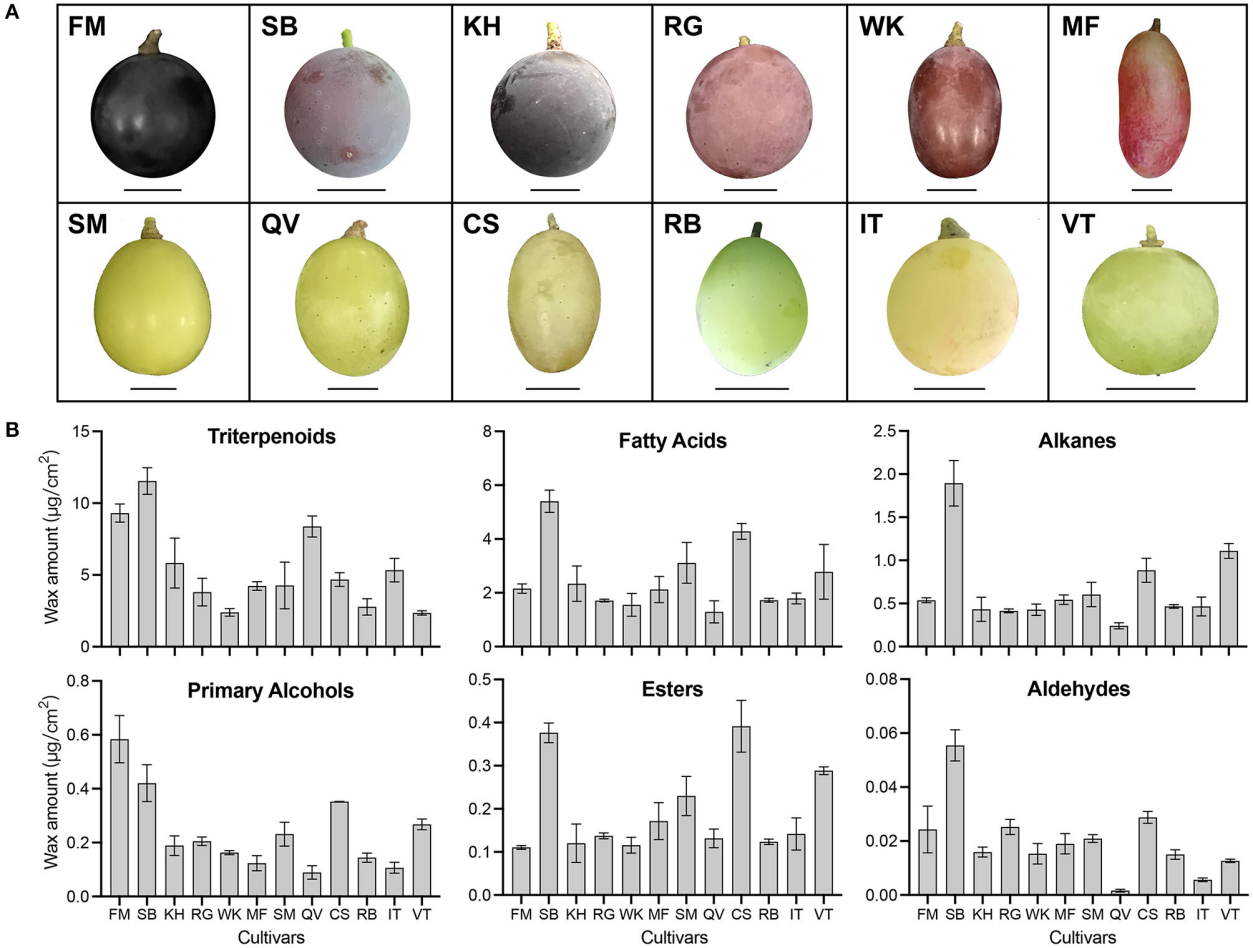
Berry morphology **(A)** and wax amount **(B)** in twelve grape cultivars. Twelve grape cultivars: KH, “Kyoho”; RG, “Red Globe”; SM, “Shine Muscat”; FM, “Fujiminori”; SB, “Summer Black”; WK, “Wink”; MF, “Manicure Finger”; QV, “Queen of Vineyard”; CS, “Centennial Seedless”; RB, “Rosario Bianco”; IT, “Italia”; VT, “Victoria”. Scale bars represent 1 cm. Error bars indicate SD from three biological replicates.

To highlight interspecific differences in the amounts of wax compounds, we investigated the cuticular wax across different cultivars (Figure 3A). The GC-MS results showed that the wax components among the mature berries of 12 cultivars were quite similar, mainly including triterpenoids and VLCFA aliphatic compounds. However, the amounts of each component varied highly among different cultivars. Abundant triterpenoids were detected in 12 grape cultivars and their concentration varied greatly, ranging from 11540.78 ± 927.59 μg/cm^2^ in “Summer Black” berries to 2356.37 ± 149.81 μg/cm^2^ in “Victoria” berries. Fatty acids, alkanes, and aldehydes were important aliphatic compounds in mature grape berries and all of these compounds showed the highest amounts in “Summer Black” but the lowest amounts in “Queen of Vineyard”. “Queen of Vineyard” berries also contained the lowest amounts of primary alcohols. “Fujiminori” berries contained the highest amounts of primary alcohols but the lowest amounts of esters. The highest concentration of esters was observed in “Centennial Seedless” berries (Figure 3B). To better understand how the cuticular wax compositions were affected by developmental stages and species, we performed a partial least squares-discriminant analysis (PLS-DA). The results showed that grape berries were clearly distinguished. Berries of three cultivars at three different developmental stages were clearly separated (Supplementary Figure 1A), whereas the separation among different cultivars was relatively smaller (Supplementary Figure 1B). These results indicate that the amounts of each wax compounds varied significantly among different cultivars and showed no correlation with berry skin color.

### Total Wax Content and Composition During Berry Development and Among Different Cultivars

**Figure 4:**
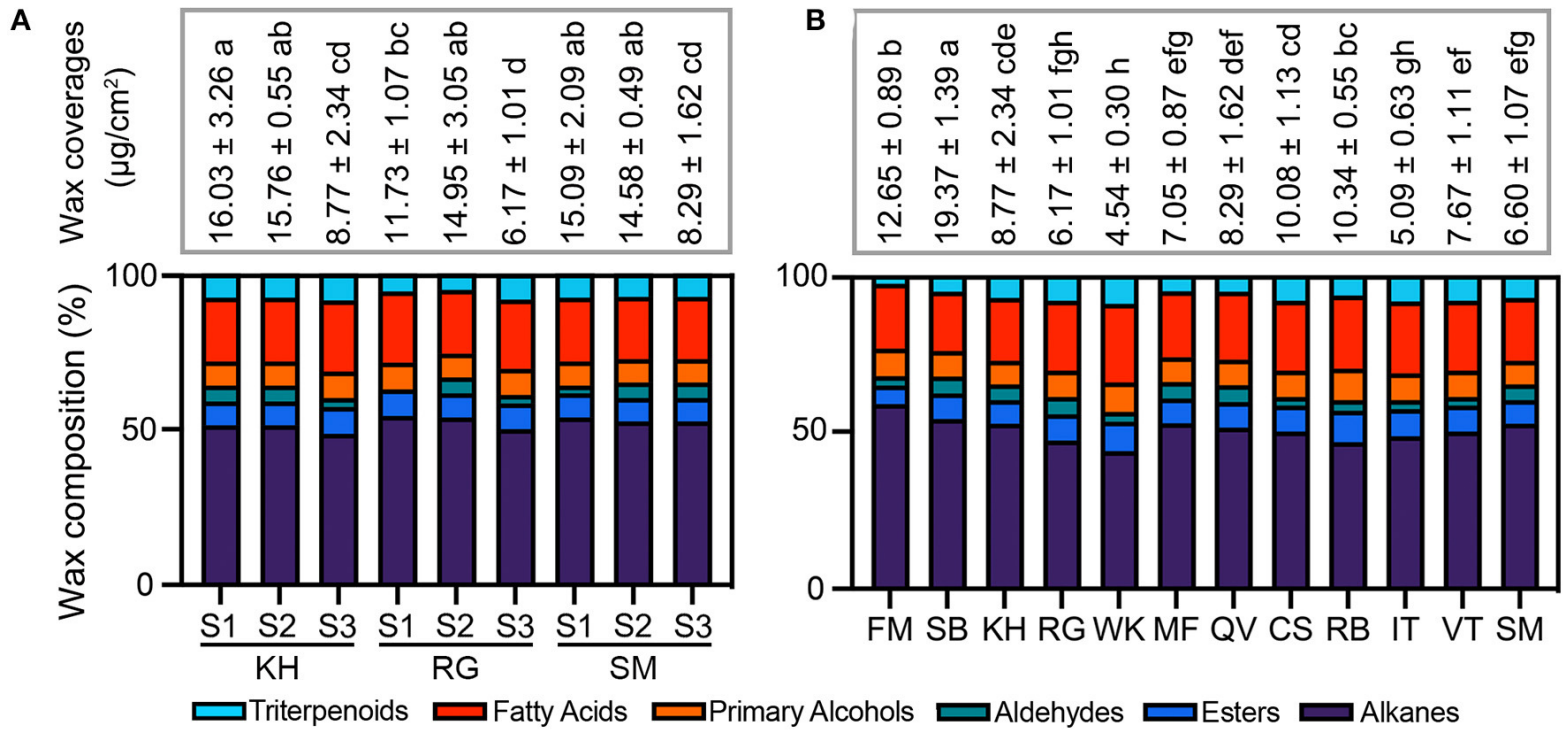
Total amount of cuticular wax and compositional proportion of compounds during berry development **(A)** and among different cultivars **(B)**. Twelve grape cultivars: KH, “Kyoho”; RG, “Red Globe”; SM, “Shine Muscat”; FM, “Fujiminori”; SB, “Summer Black”; WK, “Wink”; MF, “Manicure Finger”; QV, “Queen of Vineyard”; CS, “Centennial Seedless”; RB, “Rosario Bianco”; IT, “Italia”; VT, “Victoria”. Three developmental stages: S1, mature-green; S2, véraison; S3, maturation. Values are mean ± SD of three biological replicates. Different letters indicate significant differences at *P* < 0.05 (Student's *t*-test).

The total content and the compositional proportion of wax compounds were analyzed during berry development (Figure 4A) and among 12 grape cultivars (Figure 4B). The wax amount per surface area remained more or less constant between S1 and S2 stages, then decreased at the ripe stage (S3) in “Kyoho,” “Red Globe,” and “Shine Muscat” berries. From S1 to S3, the total wax amount decreased almost by 50% in the three cultivars. At the same development stage, no marked differences in total wax amounts among the three cultivars was detected. During the development, the compositional proportion of most compounds stayed almost stable, while that of aldehydes showed different trends among three detected varieties (gradually decreasing in “Kyoho,” firstly increasing and then decreasing in “Red Globe”; gradually increasing in “Shine Muscat”). The total wax amounts of mature fruits were highly variable among 12 grape cultivars, ranging from 4.79 μg/cm^2^ in “Wink” to 20.57 μg/cm^2^ in “Summer Black”. The most diversity of compounds was alkanes, followed by fatty acids.

### Expression Profiling of Wax-Related Genes During Berry Development

**Figure 5:**
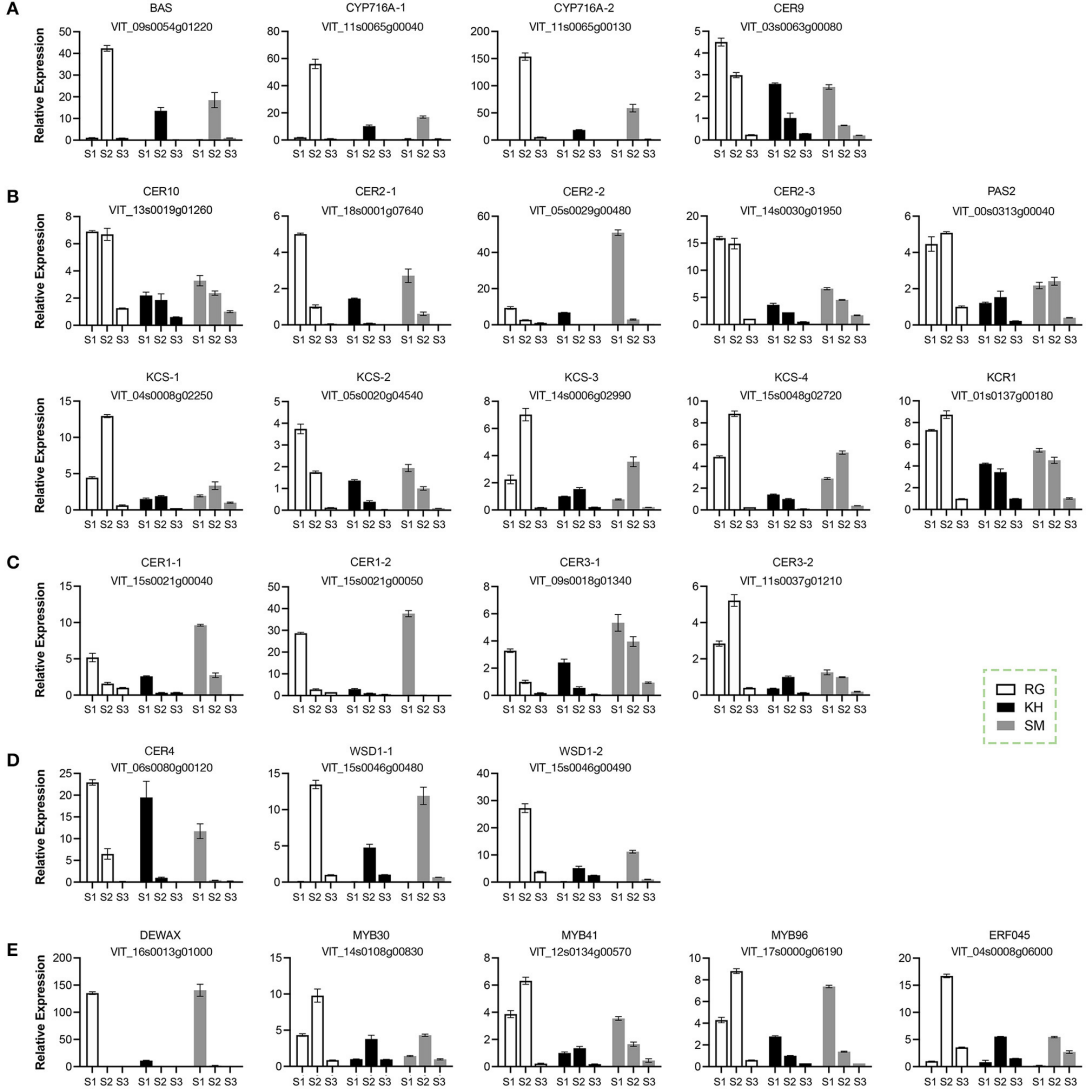
Relative expression of wax-related gene in grape berries during development. **(A)** Genes involving in the triterpenoid biosynthesis. **(B)** Genes involving in the fatty acid elongation. **(C)** Genes involving in the alkane biosynthesis. **(D)** Genes involving in the alcohol biosynthesis. **(E)** Transcription factors involving in the regulation of wax. Three grape cultivars: KH, “Kyoho”; RG, “Red Globe”; SM, “Shine Muscat”. Three developmental stages: S1, mature-green; S2, véraison; S3, maturation. Error bars indicate SD from three biological replicates.

To gain insight into the molecular basis of wax formation, 26 candidates potentially involved in the cuticular formation were selected for the gene expression analysis on the basis of previous studies conducted by Dimopoulos et al. (17). These candidates included 4 structural genes involving in triterpenoid biosynthesis (β-amyrin synthase *BAS, CYP716A, CER9*; Figure 5A); 10 structural genes involving in fatty acid elongation (*CER10, CER2*, very-long-chain hydroxy fatty acyl-CoA dehydratase *PAS2, KCS, KCR1*; Figure 5B); 7 structural genes involving in alkane (*CER1, CER3*; Figure 5C) and alcohol (*CER4, WSD1*; Figure 5D) biosynthesis; and 5 transcription factors (*DEWAX, MYB30, MYB41, MYB96, ERF045*) (Figure 5E). Overall, these genes showed two types of expression patterns during the grape berry development; one was that the expression levels were high at the early developmental stage (S1) and then gradually decreased, such as *CER9*; the other was that the expression levels were induced just before véraison and declined sharply thereafter, such as *MYB96*. All genes showed very low expression levels at the ripe stage (S3), which was consistent with the low amounts of wax compounds.

### Changes in Wax Amount and Related Genes' Expression During Post-harvest Storage

**Figure 6:**
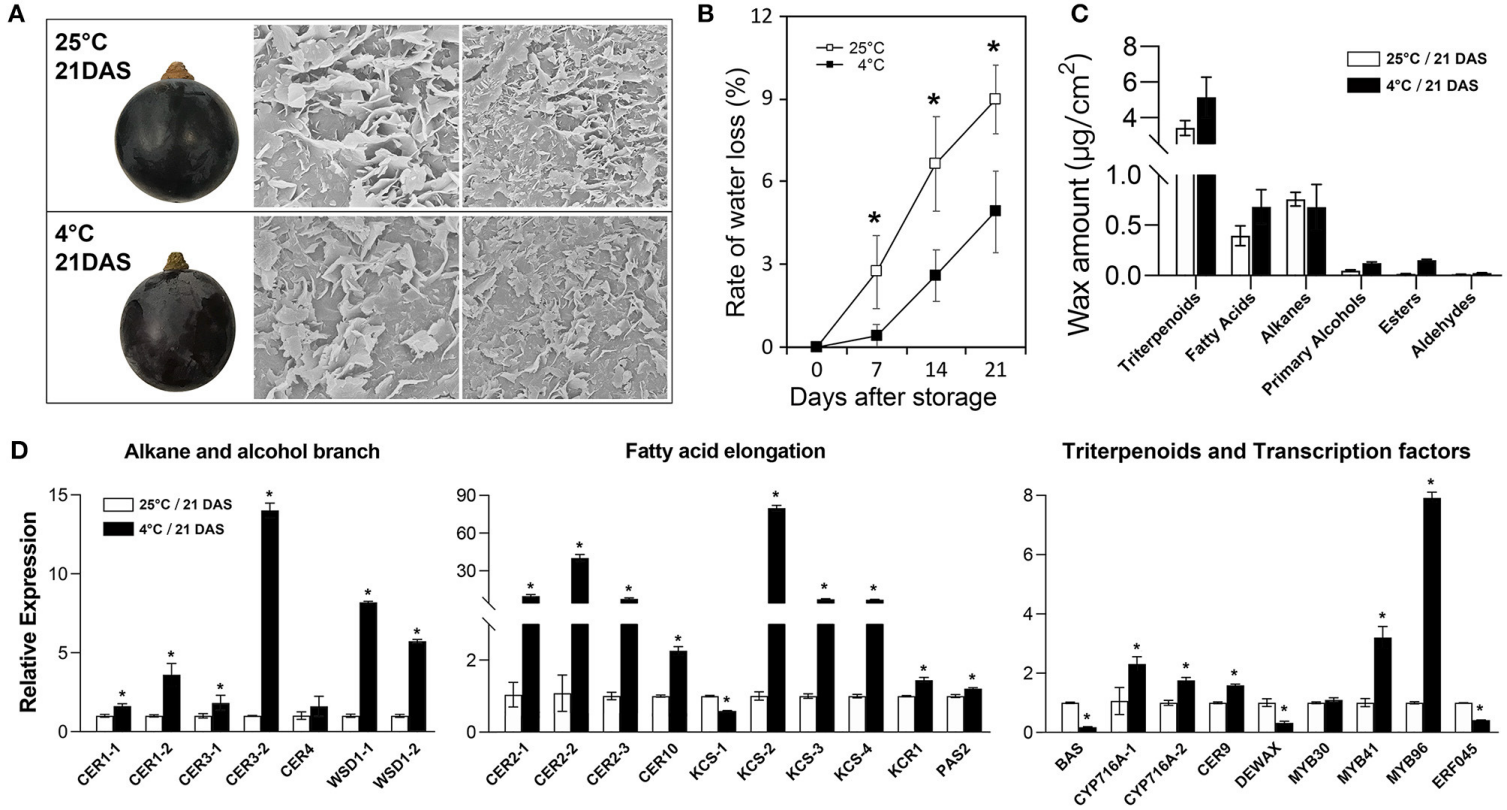
Morphological, physiological, and molecular analysis of cuticular wax in grape berries during post-harvest storage. **(A)** berry morphology and microstructure of cuticular wax; **(B)** rate of water loss; **(C)** wax amount; **(D)** relative expression of wax-related genes. DAS, days after storage. Values are mean ± SD of three biological replicates. Significant differences at the *P* < 0.05 level were indicated by * according to Student's *t*-test.

To further investigate changes in cuticular wax of grape berry during storage, berries of “Kyoho” grape were stored at room temperature (25°C) and at cold conditions (4°C) for almost 3 weeks. The Cryo-SEM results revealed a high density of platelet-like wax crystals scattered on berry surfaces and the crystal structures showed no significant difference between two storage conditions (Figure 6A). The rate of water loss under 25°C was almost two times more than that under 4°C throughout the storage process (Figure 6B). Triterpenoids remained the most abundant wax fraction and aldehydes were the less wax fraction in berries. At 21 days after storage, the amounts of wax compounds, including triterpenoids, fatty acids, primary alcohols, esters, and aldehydes, in the berries stored at 4°C were much higher than those stored at 25°C; while alkanes were the opposite (Figure 6C). We also examined the expression levels of 26 wax-related genes in the berries after storage. The results showed that the transcripts of most genes in the berries stored at 4°C were significantly higher than those stored at 25°C, such as *CER9* and *MYB96*. Several genes showed the opposite, such as *DEWAX* (Figure 6D).

## Discussion

Cuticular wax is a conspicuous white layer that is formed during fruit ontogeny (2). It determines the glossy quality of grapes and features as a protective barrier against environment (3). Therefore, characterization of cuticular wax in grape berry is of great significance. Our research provided a thorough study of cuticular wax in grape berry during berry development and post-harvest cold storage from the perspectives of crystal morphology, chemical composition, and gene expression.

### The High Density of Irregular Lamellar Crystal Structures Was Correlated With the Glaucous Appearances of Grape Berry

Cuticular waxes on fruit surface have a variety of morphological differences among species, such as rodlet and plate in bilberry (19), flattened platelet in citrus (15), platelet and rodlet in pear (12), and tubular in blueberry (20). Compared to spindly-like structures in Gewürztraminer grape berry under drought stress (17), this study found that the crystal morphology of grape epicuticular wax appeared like stacked platelets with irregular patterns (Figure 1). The inconsistency in the results may be attributed to the differences in cultivars and growth environments. In addition, compared with the incompletely covered crystals on the surface of citrus and pear (12, 15), the grape berry surface was fully covered with cuticular wax (Figure 1).

Crystal morphology and chemical composition of the plant cuticular wax are closely related. Generally, the various crystal morphologies, such as plat, platelets, rodlets, tubules, and flakes, are formed by self-assembly of waxy molecules (16). Triterpenoids and fatty acids were the major compounds of cuticular wax in grape berries (Figures 2, 3), which may be related to the lamellar crystals attached to the surface. Similarily, the platelet-like crystals in *Sedum rupestre* were characterized by high amounts of triterpenoids (21). However, the platelet-like crystals found in poaceae plants (e.g., Triticum, Zea) were generally dominated by primary alcohols (16); and the platelet-like crystals in “Newhall” orange fruits were caused by a higher proportion of aldehydes and alkanes (15). These findings indicate that similar wax structures can be formed by different chemical compositions in different species. Previous studies of *Arabidopsis* leaf and stem with glaucous phenotypes showed high wax load accompanied by high density of cuticular wax crystals (22). The glossy fruits of citrus (14), cucumber (23), and bilberry (13) showed a reduction in cuticular wax load and crystalloids. In the present study, we found that glaucous appearing “Kyoho” and “Red Globe” berries contained a highly dense cover of platelets along with lamellar-like structures, while glossy appearing “Shine Muscat” berries had limited crystals throughout the fruit development (Figure 1). Since the berries of the three cultivars showed almost similar cuticular wax load (Figure 2), the results imply that the difference between glaucous and glossy appearances of grape berry may base on the cuticular wax morphology but not the wax load. Similar findings were found in the glossy mutant of bilberry (13).

### The Accumulation Characteristic of Wax Compounds and Its Underlying Molecular Regulatory Mechanisms in Grape Berry

As previously reported (5), triterpenoids were the most abundant wax components present in berries of 12 grape cultivars (Figure 3). Similar results were also reported in blueberry (20), apple (24), peach (25), and sweet cherry (26). The dominant components in fruit wax could be alkanes in pitaya (27) and tomato (28), fatty acids in bog bilberry (19), aldehydes in citrus (15, 29), primary alcohols in pear (12), octacosanol in wheat (16), etc. Due to high amounts of triterpenoids in investigated cultivars (Figures 2, 3), the cuticular wax of grape berry could be a promising source of biological activities. In addition, primary alcohols, esters, and aldehydes were detected as being small proportions of the total wax in grape berries (Figures 2, 3) (4), in accordance with the previous findings in many other fruits, such as blueberry (20) and apple (30).

Dynamic changes in wax load during fruit development have been reported (2). The increase in wax load throughout the fruit development has been reported in blueberry (31), apple (32), pear (33) and orange fruits (29). However, as previously reported (5), wax load increased or remained constant until véraison followed by a decrease in the ripe stage, which was mainly resulted from the alteration in triterpenoids (Figures 2–4). The decrease in wax amounts was seen at the late stage of ripening (S3) when rapid growth in berry size takes place. Triterpenoids continuously accumulated in cuticular wax during fruit ripening in tomato (28), orange (24), blueberry. However, previous (5) and this study found a decrease in triterpenoids during development (Figure 2), which was also observed in sweet cherry (26). Furthermore, the amounts of each wax component varied dramatically among different cultivars (Figure 3). These findings highlight the role of genetic and environmental factors in the accumulation of cuticular wax in grape berry.

To understand cuticle biology in plant species, a range of wax-related genes have been recently identified from horticultural plants. In this study, we found that all genes showed very low expression levels at the ripe stage (S3), which was consistent with the low amounts of wax compounds (Figure 5). Similar findings were also reported in previous studies in sweet cherry (34), orange (35), and tomato (28). *MYB30* (36), *MYB41* (37), *MYB96* (9), and *ERF045* (38) were reported as positive regulator of wax biosynthesis and their expression levels were strongly correlated with *CER, KCS* and other biosynthetic genes, implying that these transcription factors were crucial in the regulation of wax synthesis in grape fruit. *DEWAX* encoding an AP2/ERF-Type transcription factor negatively regulated cuticular wax biosynthesis in *Arabidopsis* leaves and stems (10). Consistently, this study found *DEWAX* was highly expressed in berries with low content of wax stored a 25°C (Figure 6D). The extremely high levels of *DEWAX* at the green stage (S1) may repress some wax-related genes to feedback regulate wax synthesis (Figure 5). Further research will be conducted to investigate the regulatory networks of wax biosynthesis at the molecular level.

### Cuticular Wax Contributed to the Water Preservation Capacity of Grape Berries

Fruit wax has potential roles in post-harvest performance (6, 7). Previous studies have shown that the post-harvest quality and storability characteristics of fruits, such as weight loss, firmness and susceptibility to physiological disorders, are greatly affected by the chemical composition, structures and properties of the cuticle (7). It was reported that cuticular components and contents were negatively correlated with water loss and shelf life (39). Leide et al. (28) reported that high contents of alkanes of tomato fruit contributed to low weight loss. As promising sources of biological activities, triterpenoids are also of significant importance for defending against biotic and abiotic stresses. In this study, we found that cold storage significantly decelerated water loss rate, elevated wax amounts, and increased wax-related genes' expression levels in grape berries (Figure 6). These findings indicate that Cuticular wax contributed to the water preservation capacity of grape berries. The decelerated water loss rate in cold-stored berries may result from the high amounts of triterpenoids and low amounts of alkanes.

## Conclusion

In summary, this study showed that the crystal structures, compound composition and amounts, and related gene expression differed significantly in various grape cultivars during berry development and after storage. The high density of irregular lamellar crystal structures was correlated with the glaucous appearance of grape berry. The expression patterns of wax-related genes were in accordance with the accumulation of wax compounds, which underlined the crucial roles of these genes in the wax formation in grape. Furthermore, the present research demonstrated the contribution of cuticular wax to the water preservation capacity of grape berries. These findings not only facilitate a better understanding of the characteristics of cuticular wax in grape, but also shed light on the molecular basis of wax biosynthesis and regulation in grape. Future research will be conducted on exploring the roles of structure-function relationship of cuticular wax compounds and molecular regulation mechanisms of different crystal formation.

## Data Availability Statement

The original contributions presented in the study are included in the article/Supplementary Material, further inquiries can be directed to the corresponding author/s.

## Author Contributions

SL conceived and designed the research and wrote the manuscript. MZ performed the experiments and analyzed the data with help from PZ, QO-y, SL, YZ-g, HJ, and JF. RT provided technical support to analyze the GC-MS data. All authors have read and approved the manuscript.

## Funding

This study was funded by the National Natural Science Foundation of China (No. 31901991), the Natural Science Foundation of Jiangsu Province (BK20190529), the Jiangsu Agriculture Science and Technology Independent Innovation Fund (CX(21)3023), the National Key Research and Development Program of China (2019YFD1001904), the Postdoctoral Research Foundation of China (2019M651857), and the Fundamental Research Funds for the Central Universities (KYYJ202115 and KJQN202028).

## Conflict of Interest

The authors declare that the research was conducted in the absence of any commercial or financial relationships that could be construed as a potential conflict of interest.

## Publisher's Note

All claims expressed in this article are solely those of the authors and do not necessarily represent those of their affiliated organizations, or those of the publisher, the editors and the reviewers. Any product that may be evaluated in this article, or claim that may be made by its manufacturer, is not guaranteed or endorsed by the publisher.
