# Supplementary material for: Comparative Analysis of Cuticular Wax in Various Grape Cultivars During Berry Development and After Storage
Source: Front Nutr. 2021 Dec 28;8:817796. doi: 10.3389/fnut.2021.817796 (PMC8748257; doi:10.3389/fnut.2021.817796)
Supplement: Supplementary file 2 [file Data_Sheet_1.PDF]

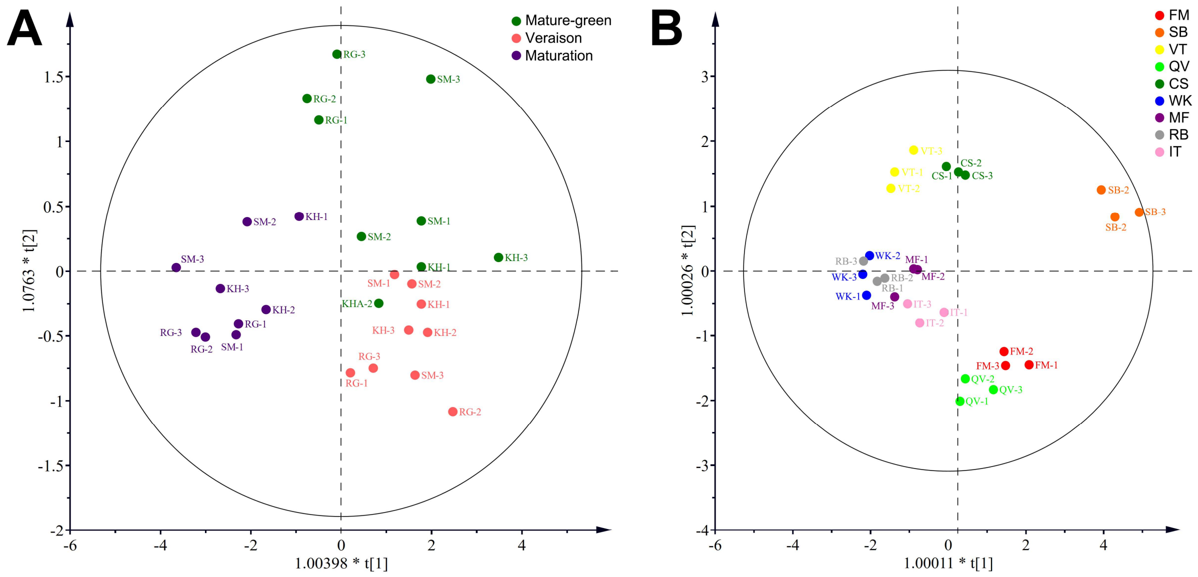

Supplemental Figure S1 Partial least squares-discriminant analysis (PLS-DA) of cuticular wax in grape cultivars during development.
